# Immune Dysregulation and Persistent Symptoms: Insights into T Cell Dynamics in Post-COVID among Athletes from the CoSmo-S Study

**DOI:** 10.1007/s10875-026-02020-2

**Published:** 2026-04-27

**Authors:** Miriam Ringleb, Daniel Alexander Bizjak, Andreas Michael Nieß, Hannah Notbohm, Hans-Georg Predel, Christian Puta, Jürgen Michael Steinacker, Manuel Widmann, Jonas Zacher, Wilhelm Bloch, Florian Javelle

**Affiliations:** 1 Department of Sports Medicine and Health Promotion, Friedrich-Schiller-University Jena, Jena, Germany; 2 Center for Interdisciplinary Prevention of Diseases related to Professional Activities, Friedrich-Schiller- University Jena, Jena, Germany; 3 NeuroPsychoImmunology Research Unit, Department for Molecular and Cellular Sports Medicine, Institute of Cardiovascular Research and Sports Medicine, German Sport University Cologne, Cologne, Germany; 4 Division of Sports and Rehabilitation Medicine, Department of Internal Medicine, Ulm University Medical Center, Ulm, Germany; 5 Department of Sports Medicine, Medical Clinic, University Hospital Tübingen, Tübingen, Germany; 6 Interfaculty Research Institute for Sports and Physical Activity, Eberhard-Karls-University of Tübingen, Tübingen, Germany; 7 Department for Molecular and Cellular Sports Medicine, Institute of Cardiovascular Research and Sports Medicine, German Sport University Cologne, Cologne, Germany; 8 Department for Preventive and Rehabilitative Sports and Performance Medicine, Institute of Cardiovascular Research and Sports Medicine, German Sport University Cologne, Cologne, Germany; 9 Department for Internal Medicine IV (Gastroenterology, Hepatology and Infectious Diseases), Jena University Hospital, Jena, Germany; 10 Center for Sepsis Control and Care (CSCC), Jena University Hospital, Friedrich-Schiller-University Jena, Jena, Germany; 11 Institute of Rehabilitation Medicine Research, University Ulm, Ulm, Germany

**Keywords:** Effector T cells, Tregs, Post-COVID condition, Athletes, Immune dysregulation

## Abstract

**Background:**

Over 10% of all SARS-CoV-2 infections lead to persistent symptoms, a condition called post-COVID condition (PCC). For elite athletes, whose performance is central, PCC poses significant challenges. It is suggested that immune cells, particularly regulatory and effector T cells, play a key role in symptom persistence nonetheless it has not yet been investigated.

**Objective:**

This study investigates immune dynamics after SARS-CoV-2 infection and assesses whether symptom persistence is accompanied by T cell dysregulation in highly trained athletes.

**Methods:**

Thirty-six highly trained athletes were included in this study after experiencing SARS-CoV-2 infection. Athletes’ data were analyzed 2–4 weeks after infection (T0) and 3–4 months later (T1). They were categorized into two groups: those with persistent symptoms (PS) and those without (SF). Their immune cell distribution was assessed via flow cytometry.

**Results:**

In the PS group, there was an increase in T helper (Th) cell 17 and Th2 cells from T0 to T1, whereas in the SF group, these cell types either decreased or remained unchanged, respectively. Furthermore, Th1 cells decreased and natural (NK) cells increased from T0 to T1 in the PS group, while no changes were observed in the SF group. No changes were observed in Tregs nor in other cell types.

**Conclusion:**

This dysregulation of the immune system toward humoral immunity indexed by a rise in Th2 and Th17 cells and a decrease in Th1 cells over time could be indicative of a possible virus persistence contributing to persistent symptoms.

**Clinical Trial Registration:**

The study has been registered in the German Clinical Trials Register (DRKS00023717).

**Graphical Abstract:**

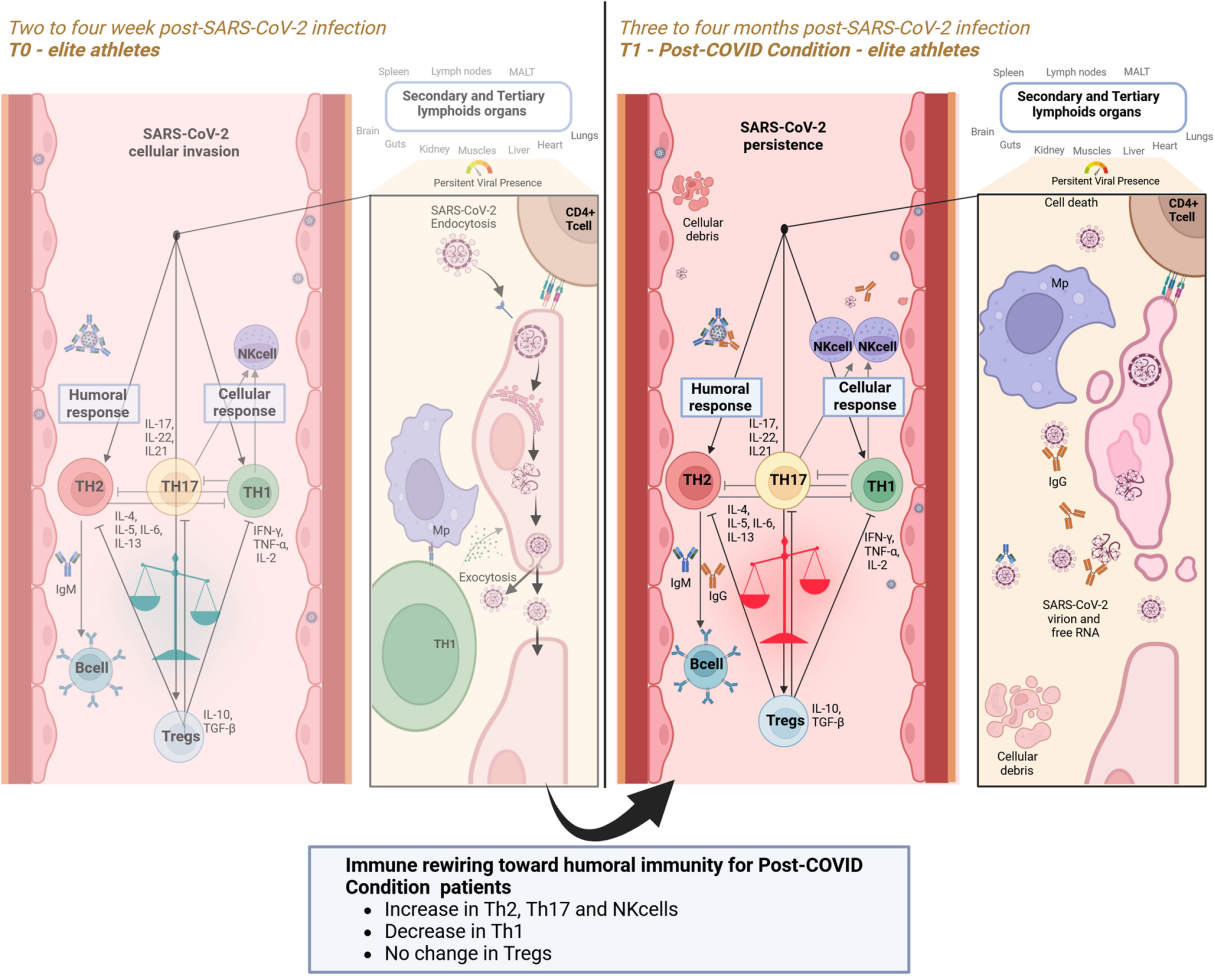

**Supplementary Information:**

The online version contains supplementary material available at 10.1007/s10875-026-02020-2.

## Introduction

Although most people have now recovered from at least one COVID-19 infection, there is a significant proportion who continue to struggle with persistent symptoms even after the acute phase of the illness. Long Covid is an infection-associated chronic condition (post-COVID condition, PCC) that occurs after severe acute respiratory syndrome coronavirus 2 (SARS-CoV-2) infection and is present for at least three months as a continuous, relapsing and remitting, or progressive disease state that affects one or more organ systems [1, 2]. Additionally, there is evidence that symptoms can also persist up to 12 months post-infection [3]. Accurately assessing the prevalence of PCC remains challenging due to the lack of a definitive diagnostic test; however, it is widely recognized that the condition affects over 10% of all SARS-CoV-2 infections, independently of fitness level [4].

A systematic review from 2021, including 39 studies investigating PCC in recovered patients, identified the most common PCC symptoms as weakness (41%), general malaise (33%), fatigue (31%), impaired concentration (26%), and breathlessness (25%) [5]. It has been suggested that patients who initially experienced severe symptoms report post-acute respiratory symptoms, such as shortness of breath or dyspnea, more frequently than those with mild or asymptomatic COVID-19 infection [6]. Furthermore, many PCC patients report diminished exercise capacity, exercise intolerance, decreased physical performance and difficulty recovering from both physical and mental exertion (post-exertional malaise, PEM) up to three months post-infection [7]. Interestingly, vaccination against SARS-CoV-2 has been shown to reduce the occurrence of certain symptoms, such as headache [8].

In elite athletes, even minimal symptom persistence can significantly impair performance [9]. However, results regarding the recovery abilities of competitive athletes are heterogeneous. While no difference in physical performance was found between competitive athletes with and without an infection in the early recovery phase of a COVID-19 infection shown by Komici et al. [10], Vollrath et al. [9] demonstrated a declined aerobic exercise capacity and symptom persistence even three months post-infection which might be linked to dysregulated continuous immune activation (e.g., virus persistence [4]). In addition, studies of elite athletes showed that six months after infection, ~ 16% of elite athletes reported a decline in performance, with concentration problems (~ 11%) and dyspnea on exertion (~ 12%) being the most common symptoms reported in the follow-up questionnaire [11]. Furthermore, six months after the initial infection, ~ 10% of elite athletes stated that their current exercise tolerance was below 70% compared to their pre-infection state [11].

The initial infection with SARS-CoV-2 can trigger a cytokine storm [12], among other immunological responses, which is thought to be associated with acute symptom severity [13]. Additionally, this cytokine storm reinforces T helper (Th) 1 cell activity, while inhibiting Th2 cells, activating macrophages, and promoting natural killer (NK) cell cytotoxicity as well as major histocompatibility complex proteins [14]. After a common virus infection, especially the rise in Th1 and NK cells ensures the fast clearance of the virus from the organism [15, 16], associated with the elimination of symptoms.

However, persistent symptoms in PCC may be associated with dysregulated Th1 and Th2 cell responses. A reduction in Th1 activity following infection, despite the persistence of viral particles or remnants, could explain ongoing immune activation and why symptoms continue beyond the acute phase. When viral particles or fragments are released into the extracellular space, they trigger the humoral immune response. This activation engages the Th2 pathway, which promotes antibody production by plasma cells and the neutralization of extracellular viruses [17].

Research on systemic inflammation in PCC also focused on regulatory T cells (Treg) and their ratio to Th cells. A scoping review conducted by Haunhorst et al. [18] suggested that Treg dysregulation can persist for at least several months post-infection. Their results, however, indicate highly heterogeneous, individual Treg dynamics following SARS-CoV-2 infection with contradictory results between studies (decrease reported by Utrero-Rico et al. [19], increase reported by Galán et al. [20]). A rise in interleukin (IL)-6, IL-4, and interferon (IFN)-γ due to the innate immune response could specifically hinder the development of Treg, while encouraging a pro-inflammatory Th17 cell profile [21, 22]. Furthermore, in severe cases of COVID-19, there has been documented evidence of an increased Th17/Treg ratio [23]. This would lead to a persistent immune response that is not sufficiently regulated by Treg [24].

These heterogeneities may also stem from differences in immune status prior to infection. As a result, it is possible that elite athletes and highly trained individuals may respond differently to such infections than other populations (e.g., variations in Treg, memory T cells, and immunosenescence). Additionally, based on the sports performed (e.g., endurance vs. combat athletes), VO_2_max and immune profiles can also greatly vary within an athlete population [25].

Therefore, this study examined the changes in cells from the innate immune system (NK cells) and adaptive immune system (e.g., Treg, Th17, Th1, Th2, naïve T cells, B cells) following SARS-CoV-2 infection in highly trained athletes. The changes in the immune cells from two to four weeks after infection (T0) to three months after T0 (T1) were compared between athletes with and without persistent symptoms. Additionally, correlational analyses between the different cell types and participant characteristics were performed to identify possible covariates. As such, the aim is to shed light on the immune cell dynamics after infection and if symptom persistence over several months is accompanied by altered immune cell proportions as well. Furthermore, a reflection on our results and the comparison with other populations is performed.

## Methods

This study is part of CoSmo-S (COVID-19 in elite sports—A multi-center cohort Study), a consortium investigating PCC in athletes [26]. The study has been registered in the German Clinical Trials Register (DRKS00023717). Ethics approval was obtained by the Ethics Committee of the Medical Faculty, University of Tübingen on 28 July 2020 (reference number: 608/2020BO1), the Ethics Committee of the University Ulm on 3 November 2020 (reference number: 408/20), and the Ethics Committee of the German Sport University Cologne on 22 June 2020 (reference number: 087/2020). The work described is carried out following the Declaration of Helsinki.

### Study Characteristics

Due to an amendment, the study characteristics can differ from the original protocol [26]. Examinations for this sub-study were performed at the University Hospital Ulm and the German Sport University Cologne, two of the 13 sports medical outpatient clinics in Germany licensed by the German Olympic Association (DOSB) for preparticipation screenings in federal squad athletes and Paralympic athletes.

**Fig. 1:**
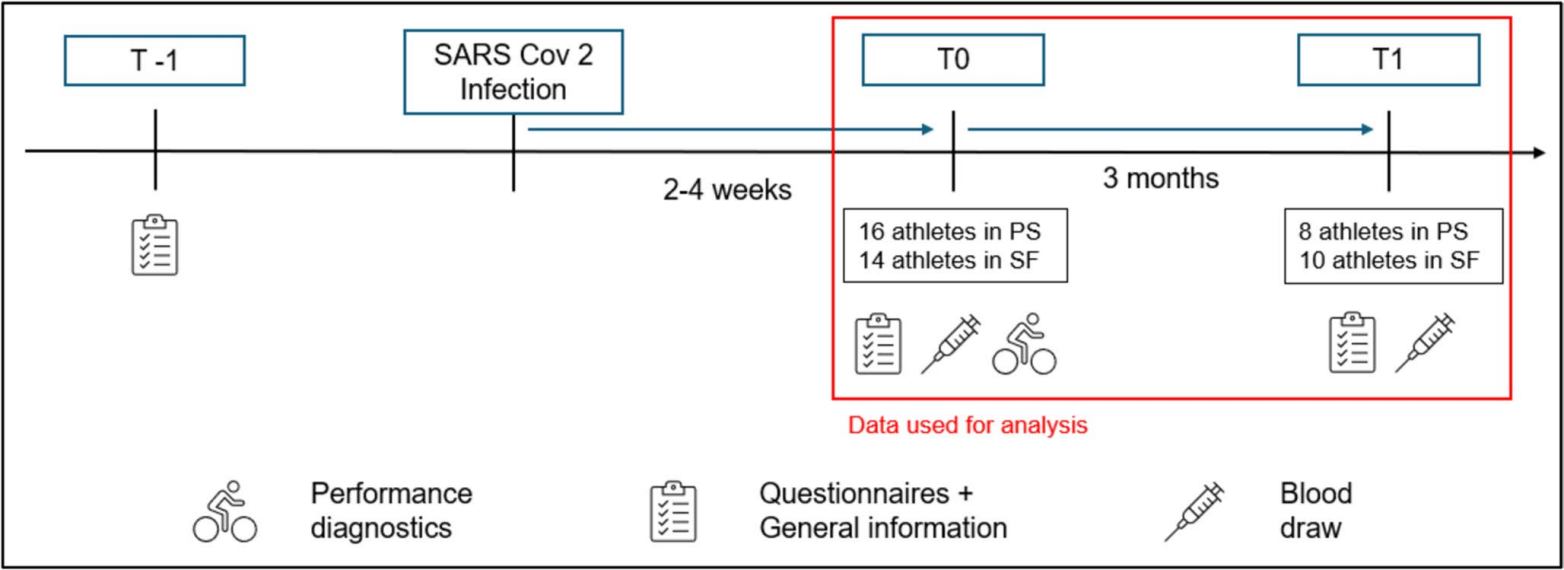
Study overview. T-1 refers to the athlete’s data taken before their SARS-CoV-2 infection, while T0 (2–4 weeks) and T1 (3 months after T0) are measurement time points after the infection. *PS* persistent symptoms, *SF* symptom free

The included participants were infected with SARS-CoV-2 two to four weeks before the examination. The infection was verified via a positive swap (polymerase chain reaction, PCR) or a positive serum SARS-CoV-2 IgG [26]. If athletes could present a positive PCR test result on the day of the examination that proves their infection at least two weeks but no more than four weeks previously, they were included in the study (T0). Blood was drawn, and information about symptoms (eight different symptom categories – more information in Supplementary Material) and general information about, e.g., their age or sports, were gathered via a diagnostic interview. The follow-up time point with blood draw and symptoms assessment was three months later, referred to as their T1. An overview of the study is displayed in Fig. 1.

### Participant Characteristics

Thirty-six athletes were included in this study. Participants were eligible for inclusion if they were 14 years or older, members of the German federal or Paralympic federal squads, who attended their annual preparticipation screening at a DOSB-licensed study center, or highly trained amateur athletes. The preparticipation screening is a sports medical routine visit for German athletes at which their health is controlled. Additionally, written informed consent was required from all participants. For those under 18, parental consent was also necessary. Exclusion criteria included a positive SARS-CoV-2 PCR test within the previous two weeks, refusal of venous blood sampling, insufficient proficiency in the German language, any acute or chronic illness deemed unsuitable by the study physician, or withdrawal of consent to participate. Detailed information on eligibility criteria can be found in the study protocol [26].

Peak oxygen consumption (VO_2_peak) was determined at T0 via cardiopulmonary exercise testing with a maximal ramp protocol. Detailed description of the procedure can be found in a previous study publication [9]. Of the 36 athletes included, only 12 had measurements at both time points, while 24 had only a T0 or T1. After inclusion, participants were divided into two groups based on their symptom persistence. Athletes with at least one persistent symptom were placed in one group (persistent symptoms - PS), while those who were asymptomatic post-infection were placed in another (symptom-free – SF). Most frequent symptoms were fatigue (45%), reduced exercise performance (45%), muscle/joint pain (27%), and headache (27%).

Participant characteristics are presented in Table 1. 84% of the athletes were not vaccinated. Among the 16% who were vaccinated, half had received two vaccination doses and the other half three vaccination doses, all with the BioNTech/Pfizer vaccine. The sports most represented were endurance related (running, riding the bike, swimming − 25%), fencing (11%), soccer (11%), judo (11%), taekwondo (11%), track and field (5.5%), rowing (5.5%), wrestling (5.5%), and others with only one athlete engaged in this sport (14.5%).

**Table 1:** Participant characteristics

Parameters	PS (*N* = 19)	SF (*N* = 17)
Sex (m/f)	12/7	10/7
Age (years)	24.9 ± 6.3 (16–44)	23.1 ± 4.2 (14–29)
BMI (kg/m^2^)	22.3 ± 2.1 (17.8–25.9)	21.9 ± 2.0 (18.7–25.3)
Weeks since infection at T0	3.3 ± 0.8 (2–4)	3.5 ± 0.8 (2–4)
VO_2_peak at T0 (ml/kg/min)	43.1 ± 8.0	43.0 ± 6.9
Training hours per week (average hours)	9.5 (5–18)	10.2 (2–14)

Note mean ± SD, range in brackets. *f* female, *m* male, *min* minute, *mil* milliliter, *N* number of athletes, *kg/m*^*2*^ kilograms per meter squared, *PS* persistent symptoms, *SF* symptom free

### Blood Processing

Peripheral blood mononuclear cells (PBMC) were isolated using Leucosep tubes. 3–8 mL of anticoagulated sample material (EDTA blood, diluted with physiological saline if necessary) were poured directly from the blood collection tube into the 12 mL Leucosep tube. Thereafter, the tube was centrifuged for 10 min at 1000 g. Then, the plasma fraction was lifted off, and the enriched cell fraction (PBMC) was harvested using a Pasteur pipette or by pouring from the Leucosep tube into a fresh centrifuge tube. The enriched cell fraction was washed thrice, firstly with 10 mL phosphate-buffered saline (PBS), centrifuged at 250 g for 10 min with brake and then again twice with 5 mL PBS. Thereafter, the cell pellet was resuspended in 2 mL freezing medium (Thermo Fisher Scientific, Germany), aliquoted into four cryotubes, and stored at -80 °C until required.

As the analysis took place at the German Sport University Cologne, the samples from Ulm were sent to Cologne. The samples were shipped on dry ice to prevent them from thawing during the 24-hour transport. On the day of the analysis, one aliquot per time point and participant was thawed in a 37 °C water bath for 90 s, then immediately diluted in 9 mL of PBS, and centrifuged at 400 g for 10 min. After discarding the supernatant, the samples were analyzed with a flow cytometer from Miltenyi Biotec (MACS Quantifyer 10).

### Flow Cytometry

Table 2 presents the cluster of differentiation (CD) enabling the identification of the different types of cells.

**Table 2:** Overview of Treg and Th panel with the corresponding CD

Treg panel		Th panel	
CD3^+^	T cells	CD3^+^	T cells
CD3^−^ CD19^+^	B cells	CD3^−^ CD16^+^	NK cells
CD3^+^ CD4^−^	Cytotoxic T cells	CD3^+^ CD4^−^	Cytotoxic T cells
CD4^+^ CD25^+^ CD127low	Tregs	CD4^+^ CD183^+^ (CXCR3^+^)	Th1 cells
CD4^+^ CD45RA^+^ CD45RO^−^	Naïve T cells	CD4^+^ CCR4^+^ (CD194^+^) CCR6^−^	Th2 cells
		CD4^+^ CCR4^+^ (CD194^+^) CCR6^+^CCR10^−^	Th17 cells

*CD* cluster of differentiation, *Th cells* T helper cells, *Treg* regulatory T cells, *CCR/CXCR* chemokine receptor

Propidium iodide was used as a viability control, while fluorescence minus controls (FMO) helped to set the gates correctly. For the FMO of the Treg panel, CD3, CD4, and CD19 were set as a backbone panel, while the corresponding panel for the Th cells was built of CD3, CD4, and CD16. Thereafter, each fluorescence marker was systematically excluded to see the spread of the other fluorochromes within the gating strategy. Isotype controls were needed to determine the non-specific binding.

For the analysis, the samples were stained with antibodies to examine Treg or Th cells. The following antibodies were used: CD3-VioBlue (REA613), CD25-VioBright 515 (REA570), CD127-PE (REA614), CD45RO-APC-Vio770 (REA611), CD45RA-PE-Vio770 (REA1047), CD4-APC-Vio770 (REA623), CD194 (CCR4)-PE-Vio770 (REA279), CD183 (CXCR3)-VioBright FITC (REA232), CD4-APC (REA623), CD196 (CCR6)-APC (REA190), CD19-VioGreen (REA675), Anti-CCR10-PE (REA326), and CD16-VioGreen (REA423) (all antibodies: Miltenyi Biotec, Germany). The gating strategies were applied as previously described in Wenning et al. [27]. The researcher responsible for carrying out the gating was blinded to the symptom status.

As this was an exploratory set-up, this gating strategy and CD also enabled us to measure memory T cells, transitional T cells, Th9, Th22, and Th1Th17 cells. These results were treated as secondary outcomes.

The samples had to consist of at least 50% living cells. This led to the exclusion of four samples at T0 (11%) and two at T1 (12%), including two participants for which both time points had to be excluded (5%).

### Statistics

As this study represents a secondary analysis of existing data, no a priori sample size calculation was performed. A post hoc power analysis was conducted based on the observed effect size to estimate the achieved statistical power. To compute the power confidence interval for each effect, an effective sample size of 12 (participants with T0 and T1) was considered too conservative, while 36 (participants included in the mixed model) was deemed too optimistic. Therefore, an effective sample size of 24 was used. A correlation of ρ = 0.7 among repeated measures at rest was assumed. For the general analysis, data from both time points could be included, 18 had only a T0 and six only a T1. At T0, 16 athletes were in the PS group and 14 were in the SF. At T1, eight were included in the PS group and ten in the SF group. Data were 3z winsorised and transformed by taking the square root. Thereafter, all data were normally distributed so that only parametric tests were used. Preliminary correlational analyses or t-tests between each cell type and participant characteristics, like sex, age, BMI, and VO_2_peak, were performed. Mixed model repeated measures ANOVAs, corrected for baseline values, were performed to test if participants with symptoms had significantly different cell counts and ratios than those without symptoms. Additionally, mixed model repeated measures ANOVAs controlling for sex, VO_2_peak, type of sports, vaccination status, dead cell ratio, and study center were performed. This mixed model approach eliminated the need for imputation, as athletes with only one measurement could be included in the analysis. Partial eta squared (*ƞ*_*p*_^*2*^) was the effect size reported for the main effects, while Cohen’s d (*d*) was used to display the effect size for post-hoc analyses either computed based on adjusted means and pooled standard deviation and based on adjusted means and the model variance (Supplementary material – Table A3). The significance level was set at *p ≤* .05. The same statistical methods were applied for the secondary outcomes of this exploratory analysis (Supplementary Material).

## Results

Correlational analyses between the different types of cells and age and BMI at baseline showed no significance, indicating no need to control for these covariates beyond baseline differences (see Supplementary Material – Figure A1).

**Fig. 2:**
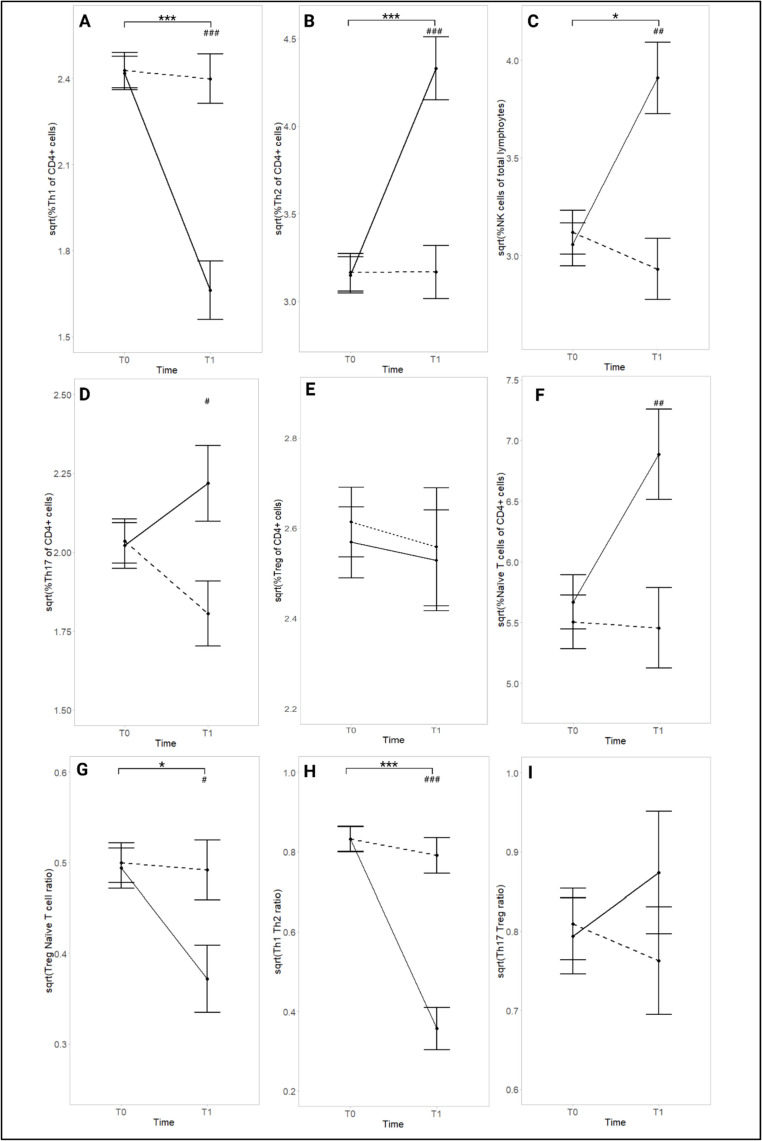
Participants’ levels of (**A**) Th1, (**B**) Th2, (**C**) NK cells, (**D**) Th17, (**E**) Treg, (**F**) Naïve T cells, (**G**) Treg/Naïve T cell ratio, (**H**) Th1/Th2 ratio, (**I**) Th17/Treg ratio from T0 to T1. Data are presented as adjusted means for baseline levels ± standard error. At T0, 16 athletes were in the PS group and 14 were in the SF. At T1, eight were included in the PS group and ten in the SF group. Only post hoc results from the time-by-group interaction are presented. Solid line: PS; dashed line: SF. Time differences are presented using the following symbols; *: *p* < .050, **: *p* < .010, ***: *p* < .001. Group differences are presented using the following symbols; #: *p* < .050, ##: *p* < .010, ###: *p* < .001

Results are displayed in Fig. 2. There was a large interaction effect for Th1 (*η*_*p*_*²*= 0.470, *p* < .001) with the PS group displaying a significant decrease from T0 to T1 (*d* = -0.92, *p* < .001, *large effect*) (Fig. 2A). A large interaction was also shown for Th2 (*η*_*p*_*²*= 0.420, *p* < .001) with the PS group showing an increase from T0 to T1 (*d* = 1.40, *p* < .001, *large effect*) (Fig. 2B). Additionally, the Th1/Th2 ratio had a large interaction effect (*η*_*p*_*²*= 0.530, *p* < .001). Athletes with symptoms displayed a decrease from T0 to T1 (*d* = -1.36, *p* < .001, *large effect*) (Fig. 2H). NK cells displayed a large interaction effect (*η*_*p*_*²*= 0.330, *p* = .001). They increased in the PS group (*d* = 1.12, *p* = .001, *large effect*) (Fig. 2C). There was a large interaction effect also for Th17 (*η*_*p*_*²*= 0.190, *p* = .023) with the PS group showing a significant increase from T0 to T1 (*d* = 0.29, *p* = .014, *small to moderate effect*) (Fig. 2D). Also, naïve T cells displayed a large interaction effect (*η*_*p*_*²*= 0.150, *p* = .039) (Fig. 2F) with the PS group showing an increase (*d* = 0.97, *p* = .013, *large effect*). Treg (Fig. 2E) and their ratio with Th17 (Fig. 2I) displayed no significant time, group, or interaction effect. However, their ratio with naïve T cells showed a large interaction effect (*η*_*p*_*²*= 0.140, *p* = .045) with the PS group displaying a significant decrease from T0 to T1 (*d* = -1.12, *p* = .010, *large effect*) (Fig. 2G).

Significant results were also shown for Th9, Th22, and Th1Th17. There was no significant interaction effect for transitional T cells, memory T cells, and B cells. As these are only secondary results obtained from the exploratory set-up of this study, further details are only provided in the Supplementary Material in Table A2. The results of the post hoc power analysis can be found in the Supplementary material in Table A2 as well. ANOVAs controlled for type of sports, sex, VO_2_peak, vaccination status, study center, and dead cell ratio did not show different results compared to controlling for cell-specific baseline differences only. Therefore, their results can be found in the supplementary material (Tables A4-A9).

## Discussion

To the best of our knowledge, this study is the first aiming to shed light on the depth immune cell profile dynamics after SARS-CoV-2 infection and to determine whether symptom persistence over several months is accompanied by altered immune cell proportions in highly trained individuals in the recovery period up to four months after SARS-CoV-2 infection. The results of this study demonstrated an increase in Th2, Th17, naïve T cells, and NK cells over time, while Th1 cells decreased from T0 to T1 in athletes with persistent symptoms. No change was observed for Treg in highly trained athletes with and without persistent symptoms.

In general, viruses like SARS-CoV-2 use a specific protein, the spike protein, to bind to receptors on the surface of host cells (e.g., ACE2 receptors for SARS-CoV-2) [28]. After attachment, the virus either fuses with the cell membrane or enters via endocytosis, releasing its genetic material (RNA in the case of SARS-CoV-2) into the host cell (see also graphical abstract – zoomed-in section at T0). The viral RNA is copied using the host’s cellular machinery. Newly produced viral RNA and proteins are packaged into virus particles. The virus exits the cell, often by budding off or causing cell lysis. Released viruses infect neighboring cells or enter the bloodstream to spread to other tissues [29].

After an acute infection, Th1 cells initially increase to combat the virus by targeting infected cells and addressing the intracellular presence of the pathogen (graphical abstract T0) [15, 16]. A cross-inhibition occurs as IFN-γ, released from Th1 cells, suppresses Th2 differentiation [30]. Furthermore, IFN-γ is activating macrophages [31], stimulating antigen presentation [32], and promoting the activation of cytotoxic CD8^+^ T cells [33]. Additionally, tumor necrosis factor (TNF)-α and IL-2 are released, leading to inflammation and recruitment of immune cells and inducing the apoptosis of infected cells [34] and the stimulation as well activation of CD8^+^ T cells and NK cells [35], respectively.

However, as soon as apoptosis and necrosis of viruses and infected cells occur, remaining fragments are detected in the extracellular matrix and the blood by Th2 cells (graphical abstract T1). They activate the humoral immune response, which supports antibody production and the neutralization of extracellular viruses [17]. This humoral immune response involves then the inhibition of the Th1 response via IL-4 and indirectly supports the differentiation of Treg by inducing a more anti-inflammatory environment [36].

The increased number of naïve T cells, that might have been pushed forward by a chronic demand, may reflect an immature or dysregulated immune response that does not meet the requirements of an effective antiviral defence. It could be assumed that it contributes to the pathophysiology of PCC, especially in chronic inflammation and autoimmune reactions.

As Th1 cells failed to clear the virus effectively, persistent symptoms arose post-infection. This scenario is accompanied by an increase in Th17 cells, which are associated with chronic inflammation, autoimmune responses, cytokine storms, and overall immune system dysregulation. Emerging evidence highlights the pivotal role of Th17 cells in COVID-19 pathogenesis [37]. These cells not only exacerbate cytokine release but also promote Th2 responses, suppress Th1 activity, and impair Treg, further disrupting immune balance [37]. Th17 cells are key drivers of inflammation and tissue damage [38], which may underline the long-term symptoms observed in PCC, such as chronic fatigue, muscle and joint pain, and respiratory issues. This highlights the complex and multifaceted nature of Th17 involvement in immune dysregulation and disease progression.

When comparing our highly trained population to other studies, similar findings can be observed. For example, Kratzer et al. [39] demonstrated a long-term decline in both innate and adaptive immune cells, accompanied by a shift from a Th1- to a Th2-dominated serum cytokine profile in PCC patients from ten weeks to ten months post-infection.

In the group of highly trained athletes with persistent symptoms, an observed increase in NK cells from T0 to T1 suggests a potential ongoing viral issue. This rise in NK cell activation might result from the diminished adaptive immune response, such as the reduction in Th1 cells, prompting the innate immune system to compensate (e.g., through NK cell activation). However, a higher number of NK cells does not necessarily equate to functional activity [40]. In PCC, the expansion of specific NK cell subtypes, such as immature or less cytotoxic NK cells, may limit their effectiveness in supporting immune defence mechanisms [40]. This could indicate a qualitative rather than quantitative deficiency, where the increased presence of NK cells does not translate to an adequately robust antiviral response.

Tsao et al. [41] compared the proportion of NK cells between PCC patients four months following infection with fully recovered individuals. Interestingly, they showed that PCC patients had significantly fewer NK cells compared to fully recovered participants, contradicting our findings and possibly showing different responses between athletes and non-trained individuals.

The observed shift in effector T cells and increase NK cells may suppress immunoregulatory mechanisms mediated by Treg, as no significant change in Treg levels was noted from T0 to T1. This dysregulated immune activation could explain the persistent symptoms seen in athletes. A scoping review conducted by Haunhorst et al. [18] suggested that Treg dysregulation can persist for at least several months post-infection. Their results, however, indicate highly heterogeneous, individual Treg dynamics following SARS-CoV-2 infection affected by factors such as age, sex, or training status. Although no firm conclusion on the direction and consistency of the Treg dynamics could be drawn, theories about their underlying biological mechanisms have been developed. For instance, the rise in IL-6 and IL-17 could specifically hinder the development of Treg and the induction of Forkhead-Box-Protein (FoxP) 3, the key transcription factor of Treg, while encouraging a pro-inflammatory Th17 cell profile [21, 22].

The reduced Th1 response may impair the immune system’s ability to effectively combat the virus or subsequent infections, further contributing to chronic inflammation. Excessive Th2 activity could also exacerbate the inflammatory state, potentially linking it to the prolonged symptoms of PCC, such as fatigue, muscle pain, and respiratory issues [42]. This imbalance highlights a scenario where the immune system, instead of resolving the infection, remains in a dysregulated and overactive state, perpetuating inflammation and tissue damage. Besides, the decrease in Th22 might indicate that the ongoing inflammation is located in the gut as Th22 cells play an important role in maintaining the barrier function of the intestinal epithelium [43]. Yet, this remains to be proven with more research on tissue-specific immune dysregulations in patients suffering from PCC.

Finally, one should recall that this dysregulation of the immune system is only one part of a larger system that also includes red blood cell function and poorer oxygen supply, which serve as an additional explanation for the PCC symptoms [44].

### Strengths and Limitations

The strengths of this study lie in linking changes in immune cells with the occurrence of symptoms in a rare population, namely, athletes. Indirect measurements of immune cells are often performed using cytokine changes. This study examined actual changes in athletes’ immune cells in relation to persistent symptoms. The results presented underscore the importance of immunological rewiring in connection with PCC and other post-viral diseases.

The presented results must be, however, interpreted in the context of their limitations. Some samples had a relatively low quality, displayed by a high percentage of dead cells. Therefore, the accepted rate of dead cells was set at 50%. To ensure, that all cell types died equally in those samples, manipulation checks were carried out beforehand between samples with high rates of dead cells (> 15%) and low rate of dead cells (< 15%). No significant difference was found.

In this study, a binary scoring system was employed to differentiate between athletes with and without persistent symptoms. Future research should consider examining symptom severity and its association with immune cell changes. Incorporating a qualitative analysis of symptom severity, alongside the quantitative findings from this study would provide a more comprehensive understanding of the underlying mechanisms.

In addition, when examining the athlete’s sport as a covariate, considerable heterogeneity within the groups was evident due to the binary classification between endurance and combat sports. However, this was the most appropriate method for categorizing the sports performed. Moreover, retrograde amnesia was not assessed in this study. However, it could have potentially influenced the athlete’s reponses to the the symptom questions. Therefore, it should be included in future studies investigating PCC symptoms.

Furthermore, a larger sample and analyses for other population groups are required in order to obtain even more generalizable results. This is especially relevant as the confidence interval for the post hoc calculated power for some immune cells is quite large (Supplementary Material Table A2). Moreover, although the effect sizes presented are high, they still have large confidence intervals, reflecting high interindividual heterogeneity (Supplementary Material Table A2).

Additionally, the viral variant could play a role and act as a further explanation for persistent symptoms in PCC patients [45]. Moreover, controlling for the severity of the infection would add additional value and explanation to the occurrence in PCC. Future studies should therefore also collect this information.

### Future Perspectives

Future research should emphasize the investigation of the interplay of immune cells and their corresponding cytokines. A change in the immune cell population does not necessarily influence the pro- and anti-inflammatory cytokines released. Special emphasis should be put on Th1-related cytokines (e.g., IFN-γ, TNF-α, IL-2), Th2-produced cytokines (e.g., IL-4, IL-5, IL-13), and cytokines released from Treg (IL-10, TGF-β) and Th17 cells (IL-17, IL-22). Especially, in the context of competitive sports, it is important to note the anti-inflammatory effects of (acute) exercise [46, 47], which offers a simple way to support immune regulation. As already mentioned in the example of NK cells, the functionality of immune cells can be reduced even though their number increases. It is, therefore, essential to also measure cytokine concentrations in future studies to find out which actual inflammatory processes are triggered by the change in immune cells.

## Conclusion

This is the first study highlighting the re-wiring of the immune system in the recovery period up to four months after SARS-CoV-2 infection in highly trained athletes. It is indexed by a rise in Th2, Th17, naïve T cells, and NK cells, a decrease in Th1 cells and no changes in Tregs from post-infection to three months later in athletes showing persistent symptoms. Our results are the first to provide a possible explanation for persistent symptoms in PCC athletes based on immune cell dynamics. These immune alterations may be a possible explanation for reduced exercise performance in athletes with PCC. Nevertheless, future studies should attempt to expand the current picture of the disease and its effects on highly trained athletes by including further measurement times post-infection and immune cell-dependent cytokine concentrations.

## Supplementary Information

Below is the link to the electronic supplementary material.

Supplementary Material 1

## Data Availability

The data that support the findings of this study are available from the corresponding author upon reasonable request.

## Abbreviations

BMI: Body mass index
CD: Cluster of differentiation
DOSB: German Olympic Association
FMO: Fluorescence minus controls
FoxP3: Forkhead-Box-Protein 3
IFN: Interferon
IL: Interleukin
NK cell: Natural killer cell
PBMC: Peripheral blood mononuclear cell
PBS: Phosphate-buffered saline
CC: Post-COVID condition
PEM: Post-exertional malaise
PS: Persistent symptoms
RNA: Ribonucleic acid
SARS-CoV-2: Severe acute respiratory syndrome coronavirus 2
SF: Symptom free
Th cell: T helper cell
TNF: Tumor necrosis factor
Treg: Regulatory T cell
VO_2_peak: Peak oxygen uptake

## References

[1] Ely EW, Brown LM, Fineberg HV. Long Covid Defined. N Engl J Med. 2024;391:1746–53. 10.1056/NEJMsb2408466. 39083764 10.1056/NEJMsb2408466 PMC11687645

[2] Soriano JB, Murthy S, Marshall JC, Relan P, Diaz JV. A clinical case definition of post-COVID-19 condition by a Delphi consensus. Lancet Infect Dis. 2022;22:e102–7. 10.1016/S1473-3099(21)00703-9. 34951953 10.1016/S1473-3099(21)00703-9 PMC8691845

[3] Babicki M, Kołat D, Kapusta J, Kałuzińska-Kołat Ż, Jankowski P, Mastalerz-Migas A, et al. Prevalence and assessment of risk factors among Polish adults with post-COVID syndrome: 12-month follow-up study. Pol Arch Intern Med. 2023. 10.20452/pamw.16512. 37338234 10.20452/pamw.16512

[4] Altmann DM, Whettlock EM, Liu S, Arachchillage DJ, Boyton RJ. The immunology of long COVID. Nat Rev Immunol [Internet]. 2023;23:618–34. 10.1038/s41577-023-00904-7. 37433988 10.1038/s41577-023-00904-7

[5] Michelen M, Manoharan L, Elkheir N, Cheng V, Dagens A, Hastie C, et al. Characterising long COVID: a living systematic review. BMJ Glob Health. 2021;6:5427. 10.1136/bmjgh-2021-005427. 10.1136/bmjgh-2021-005427 PMC8478580 34580069

[6] Merad M, Blish CA, Sallusto F, Iwasaki A. The immunology and immunopathology of COVID-19. Science (1979). 2022;375:1122–7. 10.1126/science.abm8108. 10.1126/science.abm8108 PMC12828912 35271343

[7] Ribeiro Baptista B, d’Humières T, Schlemmer F, Bendib I, Justeau G, Al-Assaad L, et al. Identification of factors impairing exercise capacity after severe COVID-19 pulmonary infection: a 3-month follow-up of prospective COVulnerability cohort. Respir Res. 2022;23:68. 10.1186/s12931-022-01977-z. 35317815 10.1186/s12931-022-01977-z PMC8938727

[8] Babicki M, Kapusta J, Pieniawska-Śmiech K, Kałuzińska-Kołat Ż, Kołat D, Mastalerz-Migas A, et al. Do COVID-19 Vaccinations Affect the Most Common Post-COVID Symptoms? Initial Data from the STOP-COVID Register–12-Month Follow-Up. Viruses. 2023;15:1370. 10.3390/v15061370. 37376668 10.3390/v15061370 PMC10304551

[9] Vollrath S, Bizjak DA, Zorn J, Matits L, Jerg A, Munk M, et al. Recovery of performance and persistent symptoms in athletes after COVID-19. PLoS ONE. 2022;17:e0277984. 10.1371/journal.pone.0277984. 36477204 10.1371/journal.pone.0277984 PMC9728914

[10] Komici K, Bianco A, Perrotta F, Dello Iacono A, Bencivenga L, D’agnano V, et al. Clinical Medicine Clinical Characteristics, Exercise Capacity and Pulmonary Function in Post-COVID-19 Competitive Athletes. J Clin Med. 2021;10:3053. 10.3390/jcm10143053. 34300219 10.3390/jcm10143053 PMC8304629

[11] Widmann M, Gaidai R, Schubert I, Grummt M, Bensen L, Kerling A, et al. COVID-19 in Female and Male Athletes: Symptoms, Clinical Findings, Outcome, and Prolonged Exercise Intolerance—A Prospective, Observational, Multicenter Cohort Study (CoSmo-S). Sports Med. 2024;54:1033–49. 10.1007/s40279-023-01976-0. 38206445 10.1007/s40279-023-01976-0 PMC11052799

[12] Hu B, Huang S, Yin L. The cytokine storm and COVID-19. J Med Virol [Internet]. 2021;93:250–6. 10.1002/jmv.26232. 32592501 10.1002/jmv.26232 PMC7361342

[13] Mehta P, McAuley DF, Brown M, Sanchez E, Tattersall RS, Manson JJ. COVID-19: consider cytokine storm syndromes and immunosuppression. The Lancet. Lancet Publishing Group. 2020;395:1033–4. 10.1016/S0140-6736(20)30628-0. 10.1016/S0140-6736(20)30628-0 PMC7270045 32192578

[14] Montazersaheb S, Hosseiniyan Khatibi SM, Hejazi MS, Tarhriz V, Farjami A, Ghasemian Sorbeni F, et al. COVID-19 infection: an overview on cytokine storm and related interventions. Virol J BioMed Cent Ltd. 2022;19. 10.1186/s12985-022-01814-1. 10.1186/s12985-022-01814-1 PMC9134144 35619180

[15] Garcia-Gasalla M, Berman-Riu M, Pons J, Rodríguez A, Iglesias A, Martínez-Pomar N, et al. Hyperinflammatory state and low T1 adaptive immune response in severe and critical acute COVID-19 patients. Front Med (Lausanne). Front Media S A. 2022;9. 10.3389/fmed.2022.828678. 10.3389/fmed.2022.828678 PMC9002349 35425776

[16] Gil-Etayo FJ, Garcinuño S, Utrero-Rico A, Cabrera-Marante O, Arroyo-Sanchez D, Mancebo E, et al. An early Th1 response is a key factor for a favorable COVID-19 evolution. Biomedicines MDPI. 2022;10. 10.3390/biomedicines10020296. 10.3390/biomedicines10020296 PMC8869678 35203509

[17] Vos Q, Lees A, Wu ZQ, Snapper CM, Mond JJ. B-cell activation by T-cell-independent type 2 antigens as an integral part of the humoral immune response to pathogenic microorganisms. Immunol Rev Blackwell Munksgaard. 2000;176:154–70. 10.1034/j.1600-065X.2000.00607.x. 10.1034/j.1600-065x.2000.00607.x 11043775

[18] Haunhorst S, Bloch W, Javelle F, Krüger K, Baumgart S, Drube S, et al. A scoping review of regulatory T cell dynamics in convalescent COVID-19 patients – indications for their potential involvement in the development of Long COVID? Front Immunol. 2022;13. 10.3389/fimmu.2022.1070994. 10.3389/fimmu.2022.1070994 PMC9792979 36582234

[19] Utrero-Rico A, Ruiz-Ruigómez M, Laguna-Goya R, Arrieta-Ortubay E, Chivite-Lacaba M, González-Cuadrado C, et al. A Short Corticosteroid Course Reduces Symptoms and Immunological Alterations Underlying Long-COVID. Biomedicines. 2021;9:1540. 10.3390/biomedicines9111540. 34829769 10.3390/biomedicines9111540 PMC8614904

[20] Galán M, Vigón L, Fuertes D, Murciano-Antón MA, Casado-Fernández G, Domínguez-Mateos S, et al. Persistent overactive cytotoxic immune response in a spanish cohort of individuals with long-COVID: identification of diagnostic biomarkers. Front Immunol. 2022;13. 10.3389/fimmu.2022.848886. 10.3389/fimmu.2022.848886 PMC8990790 35401523

[21] Bettelli E, Carrier Y, Gao W, Korn T, Strom TB, Oukka M, et al. Reciprocal developmental pathways for the generation of pathogenic effector TH17 and regulatory T cells. Nature. 2006;441:235–8. 10.1038/nature04753. 16648838 10.1038/nature04753

[22] Kappelmann N, Dantzer R, Khandaker GM. Interleukin-6 as potential mediator of long-term neuropsychiatric symptoms of COVID-19. Psychoneuroendocrinology. 2021;131:105295. 10.1016/j.psyneuen.2021.105295. 34119855 10.1016/j.psyneuen.2021.105295 PMC8172271

[23] Gutiérrez-Bautista JF, Rodriguez-Nicolas A, Rosales-Castillo A, Jimé Nez P, Garrido F, Anderson P, et al. Negative Clinical Evolution in COVID-19 Patients Is Frequently Accompanied With an Increased Proportion of Undifferentiated Th Cells and a Strong Underrepresentation of the Th1 Subset. Front Immunol [Internet]. 2020;11:596553. 10.3389/fimmu.2020.596553. 33324414 10.3389/fimmu.2020.596553 PMC7726249

[24] Sakaguchi S, Wing K, Yamaguchi T. Dynamics of peripheral tolerance and immune regulation mediated by Treg. Eur J Immunol. 2009;39:2331–6. 10.1002/eji.200939688. 19662638 10.1002/eji.200939688

[25] Beekley MD, Abe T, Kondo M, Midorikawa T, Yamauchi T. Comparison Of Normalized Maximum Aerobic Capacity And Body Composition Of Sumo Wrestlers To Athletes In Combat And Other Sports. J Sports Sci Med. 2006;05:13–20. PMC3863922 24357971

[26] Niess AM, Widmann M, Gaidai R, Gölz C, Schubert I, Castillo K, et al. COVID-19 in german competitive sports: protocol for a prospective multicenter cohort study (CoSmo-S). Int J Public Health. 2022;67. 10.3389/ijph.2022.1604414. 10.3389/ijph.2022.1604414 PMC8859834 35197815

[27] Wenning P, Kreutz T, Schmidt A, Opitz D, Graf C, Voss S, et al. Endurance Exercise Alters Cellular Immune Status and Resistin Concentrations in Men Suffering from Non-insulin-dependent Type 2 Diabetes. Exp Clin Endocrinol Diabetes. 2013;121:475–82. 10.1055/s-0033-1343395. 24026829 10.1055/s-0033-1343395

[28] Hussein HAM, Thabet AA, Wardany AA, El-Adly AM, Ali M, Hassan MEA, et al. SARS-CoV-2 outbreak: role of viral proteins and genomic diversity in virus infection and COVID-19 progression. Virol J BioMed Cent Ltd. 2024;21. 10.1186/s12985-024-02342-w. 10.1186/s12985-024-02342-w PMC10967059 38539202

[29] Wong NA, Saier MH. The sars-coronavirus infection cycle: A survey of viral membrane proteins, their functional interactions and pathogenesis. Int J Mol Sci MDPI AG. 2021;22:1–63. 10.3390/ijms22031308. 10.3390/ijms22031308 PMC7865831 33525632

[30] Zhang Y, Zhang Y, Gu W, Sun B. Th1/Th2 cell differentiation and molecular signals. 2014;15–44. 10.1007/978-94-017-9487-9_2. 10.1007/978-94-017-9487-9_2 25261203

[31] Trinchieri G. Cytokines acting on or secreted by macrophages during intracellular infection (IL-10, IL-12, IFN-γ). Curr Opin Immunol. 1997;9:17–23. 10.1016/S0952-7915(97)80154-9. 9039773 10.1016/s0952-7915(97)80154-9

[32] Zhou F. Molecular Mechanisms of IFN-γ to Up-Regulate MHC Class I Antigen Processing and Presentation. Int Rev Immunol. 2009;28:239–60. 10.1080/08830180902978120. 19811323 10.1080/08830180902978120

[33] Tau GZ, Cowan SN, Weisburg J, Braunstein NS, Rothman PB. Regulation of IFN-γ Signaling Is Essential for the Cytotoxic Activity of CD8 + T Cells. J Immunol. 2001;167:5574–82. 10.4049/jimmunol.167.10.5574. 11698428 10.4049/jimmunol.167.10.5574 PMC4416493

[34] Li M, Beg AA. Induction of Necrotic-Like Cell Death by Tumor Necrosis Factor Alpha and Caspase Inhibitors: Novel Mechanism for Killing Virus-Infected Cells. J Virol. 2000;74:7470–7. 10.1128/JVI.74.16.7470-7477.2000. 10906200 10.1128/jvi.74.16.7470-7477.2000 PMC112267

[35] Zimmerer JM, Horne PH, Fiessinger LA, Fisher MG, Pham TA, Saklayen SL, et al. Cytotoxic Effector Function of CD4-Independent, CD8 + T Cells Is Mediated by TNF-α/TNFR. Transplantation. 2012;94:1103–10. 10.1097/TP.0b013e318270f3c0. 23222736 10.1097/TP.0b013e318270f3c0 PMC3522862

[36] Opal SM, DePalo VA, Anti-Inflammatory Cytokines. Chest. 2000;117:1162–72. 10.1378/chest.117.4.1162. 10767254 10.1378/chest.117.4.1162

[37] Martonik D, Parfieniuk-Kowerda A, Rogalska M, Flisiak R. The role of th17 response in COVID-19. Cells. MDPI; 2021. 10.3390/cells10061550. 10.3390/cells10061550 PMC8235311 34205262

[38] Miossec P, Kolls JK. Targeting IL-17 and T H 17 cells in chronic inflammation. Nat Rev Drug Discov. 2012;11:763–76. 10.1038/nrd3794. 23023676 10.1038/nrd3794

[39] Kratzer B, Gattinger P, Trapin D, Ettel P, Körmöczi U, Rottal A, et al. Differential decline of SARS-CoV-2-specific antibody levels, innate and adaptive immune cells, and shift of Th1/inflammatory to Th2 serum cytokine levels long after first COVID-19. Allergy: European Journal of Allergy and Clinical Immunology. John Wiley Sons Inc. 2024;79:2482–501. 10.1111/all.16210. 10.1111/all.16210 39003594

[40] Claus M, Pieris N, Urlaub D, Bröde P, Schaaf B, Durak D, et al. Early expansion of activated adaptive but also exhausted NK cells during acute severe SARS-CoV-2 infection. Front Cell Infect Microbiol Front Media SA. 2023;13. 10.3389/fcimb.2023.1266790. 10.3389/fcimb.2023.1266790 PMC10499356 37712059

[41] Tsao T, Buck AM, Grimbert L, LaFranchi BH, Altamirano Poblano B, Fehrman EA, et al. Long COVID is associated with lower percentages of mature, cytotoxic NK cell phenotypes. J Clin Invest [Internet]. 2025;135. 10.1172/JCI188182. 10.1172/JCI188182 PMC11827890 39688913

[42] Walker JA, McKenzie ANJ. TH2 cell development and function. Nat Rev Immunol. 2018;18:121–33. 10.1038/nri.2017.118. 29082915 10.1038/nri.2017.118

[43] Chen J, Yao J. Th22 cells and the intestinal mucosal barrier. Front Immunol. 2023;14. 10.3389/fimmu.2023.1221068. 10.3389/fimmu.2023.1221068 PMC10461049 37646028

[44] Grau M, Presche A, Krüger A-L, Bloch W, Haiduk B. Red Blood Cell Morphology Is Associated with Altered Hemorheological Properties and Fatigue in Patients with Long COVID. Biology (Basel). 2024;13:948. 10.3390/biology13110948. 39596903 10.3390/biology13110948 PMC11592038

[45] Reynolds CJ, Gibbons JM, Pade C, Lin K-M, Sandoval DM, Pieper F, et al. Heterologous infection and vaccination shapes immunity against SARS-CoV-2 variants. Science. 2022 (1979);375:183–92. 10.1126/science.abm0811. 10.1126/science.abm0811 PMC10186585 34855510

[46] Ringleb M, Javelle F, Haunhorst S, Bloch W, Fennen L, Baumgart S, et al. Beyond muscles: Investigating immunoregulatory myokines in acute resistance exercise – A systematic review and meta-analysis. FASEB J. 2024;38:e23596. 10.1096/fj.202301619R. 38597350 10.1096/fj.202301619R

[47] Ringleb M, Fabritius F, Godde J, Puta C, Bloch W, Javelle F. Circulating Myokine Responses to Acute Endurance Exercise and Their Role in Immunoregulation: A Systematic Review and Meta‐Analysis. FASEB J. 2026;40(4):e71536. 10.1096/fj.202504780R 10.1096/fj.202504780R PMC12885107 41661185

