# Supplementary material for: Immune Dysregulation and Persistent Symptoms: Insights into T Cell Dynamics in Post-COVID among Athletes from the CoSmo-S Study
Source: J Clin Immunol. 2026 Apr 27;46(1):44. doi: 10.1007/s10875-026-02020-2 (PMC13128693; doi:10.1007/s10875-026-02020-2)
Supplement: Supplementary file 1 — Supplementary Material 1 [file 10875_2026_2020_MOESM1_ESM.docx]

**Supplementary material**

**Immune Dysregulation and Persistent Symptoms: Insights into T Cell Dynamics in post-COVID Among Athletes from the CoSmo-S Study**

Miriam Ringleb MA^1,2,3^, Daniel Alexander Bizjak PhD^4^, Andreas Nieß MD^5^, Hannah Notbohm PhD^6^, Georg Predel MD^7^, Christian Puta PhD^1,8,9^, Jürgen Steinacker MD, PhD^4^, Manuel Widmann MA^5^, Jonas Zacher MD^7^, Wilhelm Bloch MD^6^, Florian Javelle PhD^3^

1 Department of Sports Medicine and Health Promotion, Friedrich-Schiller-University Jena, Jena, Germany

2 Center for Interdisciplinary Prevention of Diseases related to Professional Activities, Friedrich-Schiller-University Jena, Jena, Germany

3 NeuroPsychoImmunology Research Unit, Department for Molecular and Cellular Sports Medicine, Institute of Cardiovascular Research and Sports Medicine, German Sport University Cologne, Cologne, Germany

4 Division of Sports and Rehabilitation Medicine, Ulm University Medical Center, Ulm, Germany

5 Department of Sports Medicine, Medical University Hospital Tübingen, Tübingen, Germany

6 Department for Molecular and Cellular Sports Medicine, Institute of Cardiovascular Research and Sports Medicine, German Sport University Cologne, Cologne, Germany

7 Department for Preventive and Rehabilitative Sports and Performance Medicine, Institute of Cardiovascular Research and Sports Medicine, German Sport University Cologne, Cologne, Germany

8 Department for Internal Medicine IV (Gastroenterology, Hepatology and Infectious Diseases), Jena University Hospital, Jena, Germany

9 Center for Sepsis Control and Care (CSCC), Jena University Hospital, Friedrich-Schiller-University Jena, Jena, Germany

**
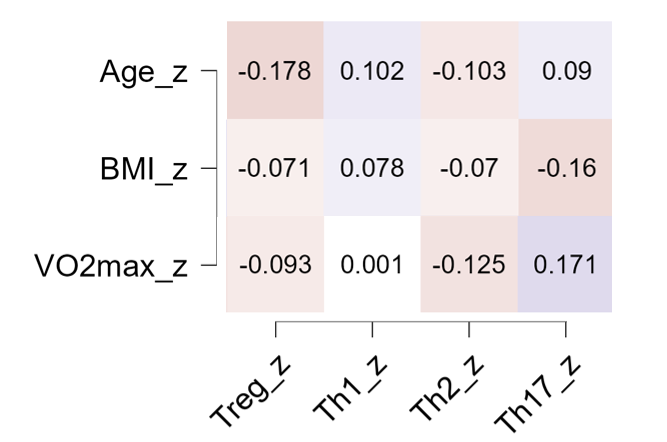
Figure A1** Heatmap displaying the correlations of the primary investigated cell types with participant characteristics at T0. *BMI* body mass index, *Th* T helper cell, *Treg* regulatory T cell, *VO2max* maximal oxygen consumption.

**
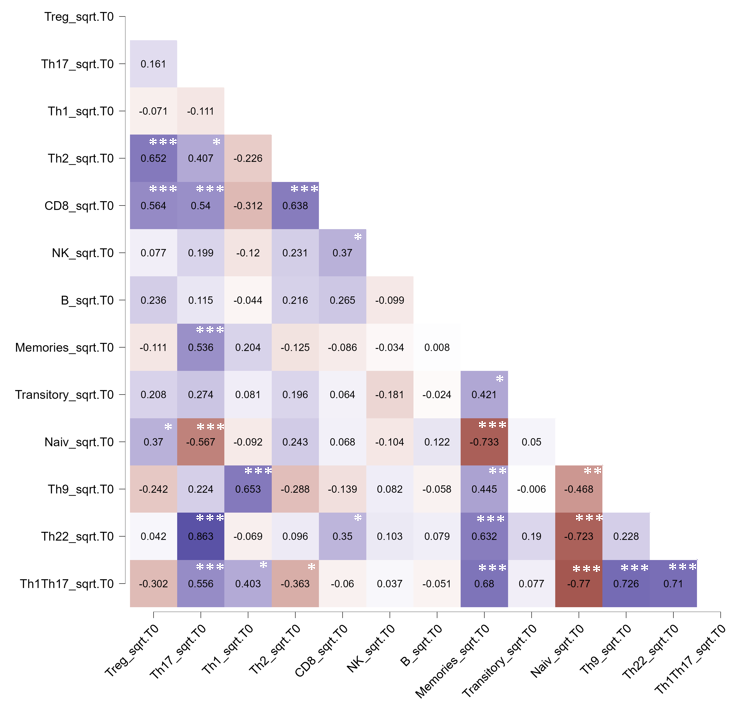
Figure A2** Heatmap displaying the correlations of the different cell types with each other at T0. Symbols indicating a significant correlation: **: p < .050, **: p < .010, ***: p < .001. CD* cluster of differentiation, *NK* natural killer cell, *sqrt* square root, *Th* T helper cell, *Treg* regulatory T cell.

**
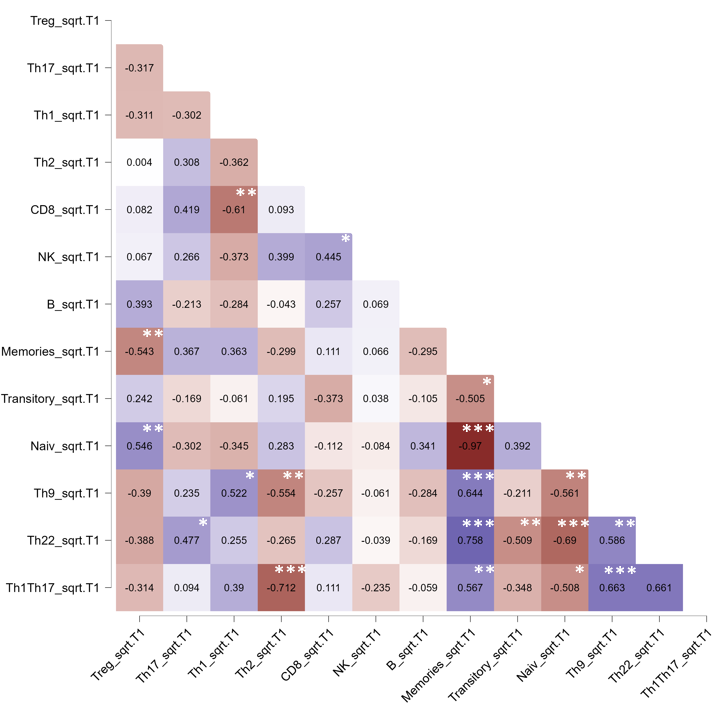
Figure A3** Heatmap displaying the correlations of the different cell types with each other at T1. Symbols indicating a significant correlation: **: p < .050, **: p < .010, ***: p < .001. CD* cluster of differentiation, *NK* natural killer cell, *sqrt* square root, *Th* T helper cell, *Treg* regulatory T cell.

***

**Table A1 Symptom occurrence**

| **Symptom category** | **Symptom-free group** | | **Persistent symptoms group** | |
| --- | --- | --- | --- | --- |
|  | **T0** | **T1** | **T0** | **T1** |
| Fatigue and performance decrease | 9% | 0% | 46% | 62% |
| Sleeping disorders | 0% | 0% | 8% | 8% |
| Neurocognitive disorders | 0% | 0% | 23% | 31% |
| Respiratory disorders | 0% | 0% | 15% | 0% |
| Autonomic/neuroendocrine disorders | 9% | 0% | 0% | 23% |
| Muscle pain | 0% | 0% | 23% | 31% |
| Psychological-related items | 0% | 0% | 8% | 0% |
| Immunological disorders | 0% | 0% | 0% | 8% |

**Statistics Secondary Results**

As this study represents a secondary analysis of existing data, no a priori sample size calculation was performed. A post hoc power analysis was conducted based on the observed effect size to estimate the achieved statistical power. To compute the power confidence interval for each effect, an effective sample size of 12 was considered too conservative, while 36 was deemed too optimistic. Therefore, an effective sample size of 24 was used. A correlation of ρ = 0.7 among repeated measures at rest was assumed. For the general analysis, for 12 participants, data from both time points could be included, while 18 had only a T0 and six only a T1. Like this, at T0, 16 athletes were in the PS group and 14 were in the SF. At T1, eight were included in the PS group and ten in the SF group. Preliminary correlational analyses or t-tests between each cell type and participant characteristics, like sex, age, BMI, and VO_2_peak, were performed. Data were 3z winsorised and transformed by taking the square root. Thereafter, all data were normally distributed so that only parametric tests were used. Mixed model repeated measures ANOVAs, corrected for baseline values, were performed to test if participants with symptoms had significantly different cell counts and ratios than those without symptoms. Additionally, mixed model repeated measures ANOVAs controlling for sex, VO_2_peak, type of sports, vaccination status, dead cell ratio, and study center were performed. This mixed model approach eliminated the need for imputation, as athletes with only one measurement could be included in the analysis. Partial eta squared (*ƞ_p_^2^*) was the effect size reported for the main effects, while Cohen's d (*d*) was used to display the effect size for post-hoc analyses either computed based on adjusted means and pooled standard deviation and based on adjusted means and the model variance (Supplementary material – Table A3). The significance level was set at *p ≤* .05. The same statistical methods were applied for the secondary outcomes of this exploratory analysis (Supplementary Material).

**Table A2** Results of the ANCOVA testing the changes in the different cell types between elite athletes with and without persistent symptoms from two to four weeks post COVID infection (T0) to three months after T0 (T1) controlling for baseline differences

| **Cell type** | **No persistent symptoms** | | **Persistent symptoms** | | **Overall** | | | **Power** |
| --- | --- | --- | --- | --- | --- | --- | --- | --- |
|  | T0_adj._ | T1_adj._ | T0_adj._ | T1_adj._ | *p* | *η_p_² (95% CI)* | *F* | *1-β (95% CI)* |
| Treg | 2.569 | 2.529 | 2.614 | 2.559 | .938 | .00 (.00 – 1.00) | F_(1,31,409)_=0.006 | 5%  (5 to 100%) |
| Th17 | 2.043 | 1.824 | 2.025 | 2.225 | .025 | .19 (.01 – 1.00) | F_(1,29,639)_=5.615 | 100%  (22.9 to 100%) |
| Th17/Treg ratio | 0.803 | 0.757 | 0.789 | 0.869 | .287 | .05 (.00 – 1.00) | F_(1,30,079)_= 1.177 | 79.2% (5 to 100%) |
| Th1 | 2.466 | 2.440 | 2.465 | 1.702 | <.001 | .47 (.22 – 1.00) | F_(1,30,735)_=21.915 | 100% (100%) |
| Th2 | 3.154 | 3.156 | 3.134 | 4.320 | <.001 | .42 (.16 – 1.00) | F_(1,32,058)_=17.342 | 100% (100%) |
| Th1/Th2 ratio | 0.846 | 0.808 | 0.855 | 0.368 | <.001 | .53 (.28 – 1.00) | F_(1,31,739)_=25.381 | 100% (100%) |
| CD8 | 6.527 | 6.540 | 6.598 | 6.643 | .929 | .00 (.00 – 1.00) | F_(1,32,292)_=0.008 | 5 to 100% |
| NK cells | 3.138 | 2.948 | 3.098 | 3.924 | .003 | .33 (.09 – 1.00) | F_(1,32,785)_=10.683 | 100% (96.7 to 100%) |
| B cells | 3.035 | 3.334 | 3.056 | 3.273 | .800 | .00 (.00 – 1.00) | F_(1,32,513)_=0.065 | 5%  (5 to 100%) |
| Memory T cells | 7.510 | 7.620 | 7.381 | 6.539 | .058 | .11 (.00 – 1.00) | F_(1,31,554)_=3.873 | 98.9%  (5 to 100%) |
| Transitional T cells | 3.092 | 3.040 | 3.069 | 3.201 | .277 | .05 (.00 – 1.00) | F_(1,31,400)_=1.222 | 79.2%  (5 to 100%) |
| Naïve T cells | 5.514 | 5.498 | 5.656 | 6.823 | .045 | .15 (.00 – 1.00) | F_(1,31,366)_=4.343 | 99.9%  (5 to 100%) |
| Treg/Naïve T cell ratio | 0.500 | 0.493 | 0.495 | 0.373 | .048 | .14 (.00 – 1.00) | F_(1,30,878)_=4.226 | 99.8%  (5 to 100%) |
| Th9 | 2.554 | 2.516 | 2.538 | 1.791 | .005 | .28 (.05 – 1.00) | F_(1,30,515)_=9.397 | 100%  (79.2 to 100%) |
| Th22 | 1.474 | 1.571 | 1.469 | 1.022 | <.001 | .39 (.14 – 1.00) | F_(1,30,188)_=16.185 | 100%  (99.8 to 100%) |
| Th1Th17 | 1.917 | 2.028 | 1.829 | 1.293 | .005 | .35 (.11 – 1.00) | F_(1,30,181)_=9.261 | 100%  (98.9 to 100%) |

Note. *CD* cluster of differentiation, *CI* confidence interval, *NK* natural killer cell, *sqrt* square root, *Th* T helper cell, *Treg* regulatory T cell.

**Table A3** Post hoc group comparisons for significant interaction effects

| **Variable** | **Time** | **Mean Diff (SF-PS)** | ***T*** | ***p* (adj.)** | **Cohen’s *d*** | **95% CI *d*** |
| --- | --- | --- | --- | --- | --- | --- |
| **Th1** | T0 | 0.001 | 0.014 | .989 | -.012 | -.76; .74 |
|  | T1 | 0.738 | 5.298 | <.001 | -1.874 | -3.08; -0.67 |
| **Th2** | T0 | 0.019 | 0.131 | .896 | -.050 | -.80; .70 |
|  | T1 | -1.164 | -4.668 | <.001 | 2.247 | .97; 3.53 |
| **Th17** | T0 | 0.018 | 0.179 | .859 | -.037 | -.79; .71 |
|  | T1 | -0.401 | -2.583 | .014 | 1.192 | .10; 2.28 |
| **Th1/Th2 Ratio** | T0 | -0.007 | -0.160 | .873 | 0.065 | -.68; .81 |
|  | T1 | 0.446 | 5.817 | <.001 | -2.786 | -4.19; -1.38 |
| **Treg/Naïve T cell ratio** | T0 | 0.005 | 0.158 | .876 | -.061 | -.81; .69 |
|  | T1 | 0.120 | 2.388 | .022 | -1.121 | -2.20; -.04 |
| **NK cells** | T0 | 0.036 | 0.227 | .822 | -.090 | -.84; .66 |
|  | T1 | -1.016 | -3.775 | <.001 | 1.810 | .61; 3.00 |
| **Naïve T cells** | T0 | 0.130 | -0.439 | .663 | .155 | -.60; .91 |
|  | T1 | 1.420 | -2.620 | .013 | 1.299 | .19; 2.40 |

Note. *CI* confidence interval, *d* Cohen’s d, *NK* natural killer cell, *PS* persistent symptoms, *SF* symptom-free *Th* T helper cell.

**Table A4** Results of the ANCOVA testing the changes in the different cell types between elite athletes with and without persistent symptoms from two to four weeks post COVID infection (T0) to three months after T0 (T1) controlling for sex

| **Cell type** | **No persistent symptoms** | | **Persistent symptoms** | | **Overall** | | | **Covariate** |
| --- | --- | --- | --- | --- | --- | --- | --- | --- |
|  | T0_adj._ | T1_adj._ | T0_adj._ | T1_adj._ | *p* | *η_p_²* | *F* | *p* |
| Treg | 2.574 | 2.577 | 2.610 | 2.553 | .772 | .003 | F_(1,30,564)_=0.085 | .594 |
| Th17 | 2.019 | 1.790 | 2.010 | 2.206 | .027 | .156 | F_(1,29,287)_=5.424 | .655 |
| Th17/Treg ratio | 0.800 | 0.753 | 0.793 | 0.870 | .298 | .037 | F_(1,29,520)_= 1.123 | .486 |
| Th1 | 2.469 | 2.439 | 2.465 | 1.694 | <.001 | .420 | F_(1,30,361)_=22.025 | .249 |
| Th2 | 3.150 | 3.159 | 3.133 | 4.331 | <.001 | .353 | F_(1,31,438)_=17.134 | .518 |
| Th1/Th2 ratio | 0.847 | 0.808 | 0.855 | 0.366 | <.001 | .445 | F_(1,31,154)_=24.975 | .691 |
| CD8 | 6.674 | 6.597 | 6.658 | 6.661 | .831 | .001 | F_(1,31,828)_=0.047 | .598 |
| NK cells | 3.142 | 2.945 | 3.098 | 3.912 | .003 | .244 | F_(1,32,221)_=10.404 | .550 |
| B cells | 3.027 | 3.345 | 3.055 | 3.296 | .811 | .002 | F_(1,32,201)_=0.058 | .170 |
| Memory T cells | 7.490 | 7.608 | 7.364 | 6.493 | .146 | .118 | F_(1,30,908)_=4.121 | .051 |
| Transitional T cells | 3.091 | 3.067 | 3.069 | 3.204 | .375 | .026 | F_(1,30,616)_=0.810 | .807 |
| Naïve T cells | 5.475 | 5.419 | 5.652 | 6.891 | .033 | .138 | F_(1,31,148)_=4.988 | .139 |
| Treg/Naïve T cell ratio | 0.502 | 0.495 | 0.494 | 0.369 | .042 | .127 | F_(1,30,820)_=4.492 | .076 |
| Th9 | 2.561 | 2.513 | 2.537 | 1.680 | .006 | .227 | F_(1,29,974)_=8.804 | .358 |
| Th22 | 1.472 | 1.570 | 1.470 | 1.024 | <.001 | .349 | F_(1,29,590)_=15.885 | .622 |
| Th1Th17 | 1.916 | 1.949 | 1.856 | 1.183 | <.001 | .319 | F_(1,31,952)_=14.952 | .967 |

Note. *CD* cluster of differentiation, *NK* natural killer cell, *sqrt* square root, *Th* T helper cell, *Treg* regulatory T cell.

**Table A5** Results of the ANCOVA testing the changes in the different cell types between elite athletes with and without persistent symptoms from two to four weeks post COVID infection (T0) to three months after T0 (T1) controlling for type of sports

| **Cell type** | **No persistent symptoms** | | **Persistent symptoms** | | **Overall** | | | **Covariate** |
| --- | --- | --- | --- | --- | --- | --- | --- | --- |
|  | T0_adj._ | T1_adj._ | T0_adj._ | T1_adj._ | *p* | *η_p_²* | *F* | *p* |
| Treg | 2.579 | 2.627 | 2.632 | 2.540 | .515 | .017 | F_(1,25,463)_=0.437 | .302 |
| Th17 | 2.005 | 1.533 | 1.994 | 2.175 | <.001 | .369 | F_(1,24,705)_=14.449 | .346 |
| Th17/Treg ratio | 0.793 | 0.613 | 0.796 | 0.855 | .025 | .189 | F_(1,24,617)_= 5.724 | .716 |
| Th1 | 2.485 | 2.420 | 2.459 | 1.721 | <.001 | .368 | F_(1,25,198)_=14.671 | .358 |
| Th2 | 3.076 | 2.991 | 3.098 | 4.269 | <.001 | .359 | F_(1,26,140)_=14.652 | .887 |
| Th1/Th2 ratio | 0.865 | 0.851 | 0.851 | 0.399 | <.001 | .403 | F_(1,26,432)_=17.879 | .184 |
| CD8 | 6.552 | 6.706 | 6.623 | 6.615 | .687 | .006 | F_(1,26,289)_=0.166 | .986 |
| NK cells | 3.064 | 3.007 | 3.055 | 3.928 | .006 | .251 | F_(1,26,228)_=8.781 | .465 |
| B cells | 3.059 | 3.527 | 3.077 | 3.323 | .561 | .013 | F_(1,26,638)_=0.346 | .627 |
| Memory T cells | 7.555 | 7.652 | 7.454 | 6.498 | .075 | .118 | F_(1,25,839)_=3.443 | .160 |
| Transitional T cells | 3.058 | 3.060 | 3.035 | 3.167 | .514 | .017 | F_(1,25,045)_=0.439 | .759 |
| Naïve T cells | 5.370 | 5.237 | 5.560 | 6.845 | .045 | .145 | F_(1,26,058)_=4.414 | .149 |
| Treg/Naïve T cell ratio | 0.513 | 0.537 | 0.505 | 0.378 | .026 | .179 | F_(1,25,865)_=5.617 | .052 |
| Th9 | 2.586 | 2.395 | 2.569 | 1.722 | .044 | .155 | F_(1,24,669)_=4.516 | .987 |
| Th22 | 1.447 | 1.431 | 1.443 | 0.993 | .004 | .286 | F_(1,24,413)_=9.854 | .397 |
| Th1Th17 | 1.919 | 1.884 | 1.864 | 1.158 | .003 | .283 | F_(1,26,481)_=10.465 | .399 |

Note. *CD* cluster of differentiation, *NK* natural killer cell, *sqrt* square root, *Th* T helper cell, *Treg* regulatory T cell.

**Table A6** Results of the ANCOVA testing the changes in the different cell types between elite athletes with and without persistent symptoms from two to four weeks post COVID infection (T0) to three months after T0 (T1) controlling for VO_2_peak

| **Cell type** | **No persistent symptoms** | | **Persistent symptoms** | | **Overall** | | | **Covariate** |
| --- | --- | --- | --- | --- | --- | --- | --- | --- |
|  | T0_adj._ | T1_adj._ | T0_adj._ | T1_adj._ | *p* | *η_p_²* | *F* | *p* |
| Treg | 2.570 | 2.577 | 2.611 | 2.564 | .794 | .002 | F_(1,30,382)_=0.069 | .843 |
| Th17 | 2.023 | 1.793 | 2.007 | 2.200 | .029 | .154 | F_(1,29,100)_=5.300 | .783 |
| Th17/Treg ratio | 0.804 | 0.758 | 0.789 | 0.867 | .298 | .037 | F_(1,29,353)_= 1.121 | .881 |
| Th1 | 2.472 | 2.439 | 2.467 | 1.673 | <.001 | .435 | F_(1,30,402)_=23.364 | .109 |
| Th2 | 3.150 | 3.156 | 3.132 | 4.339 | <.001 | .357 | F_(1,31,338)_=17.376 | .549 |
| Th1/Th2 ratio | 0.847 | 0.807 | 0.855 | 0.361 | <.001 | .449 | F_(1,31,091)_=25.364 | .483 |
| CD8 | 6.673 | 6.601 | 6.659 | 6.659 | .849 | .001 | F_(1,31,594)_=0.037 | .792 |
| NK cells | 3.141 | 2.949 | 3.099 | 3.903 | .003 | .237 | F_(1,32,091)_=9.974 | .584 |
| B cells | 3.026 | 3.337 | 3.052 | 3.336 | .936 | .000 | F_(1,32,192)_=0.007 | .102 |
| Memory T cells | 7.7482 | 7.575 | 7.378 | 6.475 | .052 | .101 | F_(1,30,667)_=3.443 | .618 |
| Transitional T cells | 3.090 | 3.063 | 3.067 | 3.222 | .307 | .034 | F_(1,30,826)_=1.079 | .260 |
| Naïve T cells | 5.488 | 5.459 | 5.634 | 6.906 | .035 | .137 | F_(1,30,729)_=4.893 | .686 |
| Treg/Naïve T cell ratio | 0.501 | 0.492 | 0.496 | 0.369 | .047 | .125 | F_(1,30,161)_=4.297 | .629 |
| Th9 | 2.560 | 2.517 | 2.539 | 1.673 | .005 | .232 | F_(1,29,827)_=9.017 | .461 |
| Th22 | 1.475 | 1.573 | 1.468 | 1.018 | <.001 | .352 | F_(1,29,487)_=15.998 | .711 |
| Th1Th17 | 1.917 | 1.952 | 1.855 | 1.176 | <.001 | .337 | F_(1,32,032)_=15.258 | .607 |

Note. *CD* cluster of differentiation, *NK* natural killer cell, *sqrt* square root, *Th* T helper cell, *Treg* regulatory T cell.

**Table A7** Results of the ANCOVA testing the changes in the different cell types between elite athletes with and without persistent symptoms from two to four weeks post COVID infection (T0) to three months after T0 (T1) controlling for vaccination status

| **Cell type** | **No persistent symptoms** | | **Persistent symptoms** | | **Overall** | | | **Covariate** |
| --- | --- | --- | --- | --- | --- | --- | --- | --- |
|  | T0_adj._ | T1_adj._ | T0_adj._ | T1_adj._ | *p* | *η_p_²* | *F* | *p* |
| Treg | 2.562 | 2.569 | 2.608 | 2.610 | .984 | .000 | F_(1,30,131)_=0.000 | .291 |
| Th17 | 2.026 | 1.800 | 2.006 | 2.184 | .038 | .142 | F_(1,28,649)_=4.738 | .579 |
| Th17/Treg ratio | 0.811 | 0.770 | 0.787 | 0.837 | .445 | .164 | F_(1,29,169)_= 5.724 | .181 |
| Th1 | 2.458 | 2.429 | 2.462 | 1.751 | <.001 | .373 | F_(1,29,938)_=17.844 | .175 |
| Th2 | 3.161 | 3.163 | 3.136 | 4.280 | <.001 | .326 | F_(1,30,913)_=14.934 | .574 |
| Th1/Th2 ratio | 0.843 | 0.804 | 0.854 | 0.388 | <.001 | .407 | F_(1,31,095)_=21.359 | .386 |
| CD8 | 6.690 | 6.617 | 6.664 | 6.545 | .903 | .000 | F_(1,32,162)_=0.015 | .115 |
| NK cells | 3.132 | 2.940 | 3.096 | 3.969 | .002 | .252 | F_(1,32,056)_=10.795 | .578 |
| B cells | 3.038 | 3.337 | 3.057 | 3.251 | .755 | .003 | F_(1,31,727)_=0.099 | .812 |
| Memory T cells | 7.448 | 7.560 | 7.367 | 6.720 | .140 | .070 | F_(1,30,597)_=2.294 | .108 |
| Transitional T cells | 3.086 | 3.062 | 3.068 | 3.232 | .304 | .035 | F_(1,30,229)_=1.095 | .468 |
| Naïve T cells | 5.527 | 5.475 | 5.647 | 6.628 | .094 | .089 | F_(1,30,637)_=2.986 | .117 |
| Treg/Naïve T cell ratio | 0.496 | 0.590 | 0.494 | 0.397 | .124 | .077 | F_(1,30,130)_=2.507 | .093 |
| Th9 | 2.543 | 2.504 | 2.533 | 1.761 | .014 | .190 | F_(1,29,393)_=6.908 | .283 |
| Th22 | 1.479 | 1.581 | 1.468 | 0.999 | <.001 | .366 | F_(1,29,076)_=16.813 | .390 |
| Th1Th17 | 1.919 | 1.952 | 1.857 | 1.154 | <.001 | .324 | F_(1,32,269)_=15.482 | .537 |

Note. *CD* cluster of differentiation, *NK* natural killer cell, *sqrt* square root, *Th* T helper cell, *Treg* regulatory T cell.

**Table A8** Results of the ANCOVA testing the changes in the different cell types between elite athletes with and without persistent symptoms from two to four weeks post COVID infection (T0) to three months after T0 (T1) controlling for study center

| **Cell type** | **No persistent symptoms** | | **Persistent symptoms** | | **Overall** | | | **Covariate** |
| --- | --- | --- | --- | --- | --- | --- | --- | --- |
|  | T0_adj._ | T1_adj._ | T0_adj._ | T1_adj._ | *p* | *η_p_²* | *F* | *p* |
| Treg | 2.573 | 2.566 | 2.617 | 2.549 | .765 | .003 | F_(1,30,139)_=0.091 | .798 |
| Th17 | 2.034 | 1.783 | 2.005 | 2.192 | .024 | .164 | F_(1,29,124)_=5.699 | .533 |
| Th17/Treg ratio | 0.811 | 0.753 | 0.787 | 0.859 | .278 | .040 | F_(1,29,431)_= 1.224 | .533 |
| Th1 | 2.463 | 2.458 | 2.455 | 1.720 | <.001 | .412 | F_(1,29,737)_=20.826 | .560 |
| Th2 | 3.162 | 3.111 | 3.156 | 4.272 | <.001 | .349 | F_(1,31,127)_=16.519 | .472 |
| Th1/Th2 ratio | 0.842 | 0.831 | 0.845 | 0.397 | <.001 | .433 | F_(1,30,882)_=23.631 | .264 |
| CD8 | 6.688 | 6.470 | 6.708 | 6.559 | .853 | .001 | F_(1,31,740)_=0.035 | .194 |
| NK cells | 3.140 | 2.937 | 3.102 | 3.911 | .003 | .244 | F_(1,31,624)_=10.204 | .886 |
| B cells | 3.042 | 3.277 | 3.079 | 3.218 | .772 | .003 | F_(1,31,512)_=0.086 | .489 |
| Memory T cells | 7.501 | 7.485 | 7.423 | 6.370 | .042 | .128 | F_(1,30,735)_=4.514 | .173 |
| Transitional T cells | 3.091 | 3.071 | 3.068 | 3.206 | .377 | .026 | F_(1,30,212)_=0.806 | .911 |
| Naïve T cells | 5.461 | 5.555 | 5.587 | 7.027 | .027 | .148 | F_(1,30,925)_=5.383 | .176 |
| Treg/Naïve T cell ratio | 0.502 | 0.486 | 0.500 | 0.361 | .038 | .135 | F_(1,30,170)_=4.699 | .282 |
| Th9 | 2.554 | 2.522 | 2.535 | 1.699 | .007 | .225 | F_(1,29,426)_=8.524 | .922 |
| Th22 | 1.478 | 1.569 | 1.467 | 1.017 | <.001 | .347 | F_(1,29,475)_=15.655 | .601 |
| Th1Th17 | 1.927 | 1.880 | 1.880 | 1.110 | <.001 | .339 | F_(1,31,744)_=16.304 | .116 |

Note. *CD* cluster of differentiation, *NK* natural killer cell, *sqrt* square root, *Th* T helper cell, *Treg* regulatory T cell.

**Table A9** Results of the ANCOVA testing the changes in the different cell types between elite athletes with and without persistent symptoms from two to four weeks post COVID infection (T0) to three months after T0 (T1) controlling for dead cell percentage (binary system with below 15% and above 15% of dead cells)

| **Cell type** | **No persistent symptoms** | | **Persistent symptoms** | | **Overall** | | | **Covariate** |
| --- | --- | --- | --- | --- | --- | --- | --- | --- |
|  | T0_adj._ | T1_adj._ | T0_adj._ | T1_adj._ | *p* | *η_p_²* | *F* | *p* |
| Treg | 2.573 | 2.566 | 2.617 | 2.549 | .765 | .003 | F_(1,30,139)_=0.091 | .798 |
| Th17 | 2.034 | 1.783 | 2.005 | 2.192 | .024 | .164 | F_(1,29,124)_=5.699 | .533 |
| Th17/Treg ratio | 0.811 | 0.753 | 0.787 | 0.859 | .278 | .040 | F_(1,29,431)_= 1.224 | .533 |
| Th1 | 2.463 | 2.458 | 2.455 | 1.720 | <.001 | .412 | F_(1,29,737)_=20.826 | .560 |
| Th2 | 3.162 | 3.111 | 3.156 | 4.272 | <.001 | .347 | F_(1,31,127)_=16.519 | .472 |
| Th1/Th2 ratio | 0.842 | 0.831 | 0.845 | 0.397 | <.001 | .433 | F_(1,30,882)_=23.631 | .264 |
| CD8 | 6.688 | 6.470 | 6.708 | 6.559 | .853 | .026 | F_(1,31,740)_=0.853 | .194 |
| NK cells | 3.140 | 2.937 | 3.102 | 3.911 | .003 | .244 | F_(1,31,624)_=10.204 | .886 |
| B cells | 3.042 | 3.277 | 3.079 | 3.218 | .772 | .003 | F_(1,31,512)_=0.086 | .489 |
| Memory T cells | 7.501 | 7.485 | 7.423 | 6.370 | .042 | .128 | F_(1,30,735)_=4.514 | .173 |
| Transitional T cells | 3.091 | 3.071 | 3.068 | 3.206 | .377 | .026 | F_(1,30,212)_=0.806 | .911 |
| Naïve T cells | 5.461 | 5.555 | 5.587 | 7.027 | .027 | .148 | F_(1,30,925)_=5.383 | .176 |
| Treg/Naïve T cell ratio | 0.502 | 0.486 | 0.500 | 0.361 | .038 | .135 | F_(1,30,170)_=4.699 | .282 |
| Th9 | 2.554 | 2.522 | 2.535 | 1.699 | .007 | .225 | F_(1,29,426)_=8.524 | .922 |
| Th22 | 1.478 | 1.569 | 1.467 | 1.017 | <.001 | .347 | F_(1,29,475)_=15.655 | .601 |
| Th1Th17 | 1.927 | 1.880 | 1.880 | 1.110 | <.001 | .339 | F_(1,31,744)_=16.304 | .116 |

Note. *CD* cluster of differentiation, *NK* natural killer cell, *sqrt* square root, *Th* T helper cell, *Treg* regulatory T cell.
